# Pre- and Post-Harvest Conditions Affect Polyphenol Content in Strawberry (*Fragaria* × *ananassa*)

**DOI:** 10.3390/plants11172220

**Published:** 2022-08-27

**Authors:** Ryohei Koyama, Misaki Ishibashi, Itsuko Fukuda, Akitoshi Okino, Ro Osawa, Yuichi Uno

**Affiliations:** 1 Department of Bioresource Science, Graduate School of Agricultural Science, Kobe University, 1-1 Rokkodai, Nada-ku, Kobe 657-8501, Japan; 2 Department of Agrobioscience, Graduate School of Agricultural Science, Kobe University, 1-1 Rokkodai, Nada-ku, Kobe 657-8501, Japan; 3 Research Center for Food Safety and Security, Graduate School of Agricultural Science, Kobe University, 1-1 Rokkodai, Nada-ku, Kobe 657-8501, Japan; 4 FIRST, Tokyo Institute of Technology, J2-32, 4259 Nagatsuta, Midori-ku, Yokohama 226-8502, Japan

**Keywords:** strawberry, functional food, total polyphenol, cold stress, post-harvest

## Abstract

The strawberry fruit contains abundant polyphenols, such as anthocyanins, flavan-3-ol, and ellagitannin. Polyphenol enrichment improves the quality of strawberries and leads to a better understanding of the polyphenol induction process. We measured the total polyphenol content of strawberry fruits under different growth conditions, developmental stages, and treatment conditions during pre-harvest and post-harvest periods. High fruit polyphenol content was observed in cold treatment, which was selected for further analysis and optimization. A transcriptome analysis of cold-treated fruits suggested that the candidate components of polyphenols may exist in the phenylpropanoid pathway. Coverage with a porous film bag excluded the effects of drought stress and produced polyphenol-rich strawberry fruits without affecting quality or quantity. The degree of stress was assessed using known stress indicators. A rapid accumulation of abscisic acid was followed by an increase in superoxide dismutase and DPPH (2,2-Diphenyl-1-picrylhydrazyl) activity, suggesting that the strawberry fruits responded to cold stress immediately, reaching the climax at around 6 days, a trend consistent with that of polyphenol content. These findings enhance our understanding of the mechanism of post-harvest polyphenol accumulation and the value of strawberries as a functional food.

## 1. Introduction

Plants can produce phenolic compounds as natural pesticides against biotic stress or as protective materials against abiotic stress during the growth period [1,2,3,4,5]. In addition, polyphenol content can be altered by changes in post-harvest conditions and treatments [6,7]. The search for environmental conditions that enhance polyphenols leads to an understanding of induction mechanisms. Although there are a variety of fruit trees that bear fruits high in polyphenols, they are difficult to handle as experimental materials because it takes more than several years from the juvenile phase to the reproductive phase. Strawberry (*Fragaria* × *ananassa*), in contrast, is a herbaceous plant with high polyphenolic fruits and takes a shorter time to ripen, allowing it to be easily handled for experimental purposes. With the release of the strawberry genome sequence [8], the infrastructure for molecular biological analysis has been established. Strawberry is also an important horticultural crop worldwide. The strawberry fruit contains various useful ingredients, such as sugars, vitamins, minerals, and non-nutritive bioactive compounds, including flavonoids, anthocyanins, and phenolic acids. All these compounds exert a synergistic and cumulative positive effect on health and disease prevention in humans [9]. Polyphenols are one of the most representative health-related components of strawberries. Polyphenols modulate factors such as gene expression, antioxidant function, and detoxification, and have anti-inflammatory and anticancer activity [4,5]. Researchers have reported the antioxidant activities of strawberry polyphenols, such as anthocyanins, flavan-3-ol, and ellagitannin [10]. Uno et al. [11] reported that strawberry polyphenols have the potential to inhibit histidine decarboxylase activity, which is associated with allergy and other biological reactions in the human body. An increase in the polyphenol content of strawberry enhances the fruit’s quality from the perspective of consumer health.

Two methods are available to produce polyphenol-rich strawberries: breeding new varieties or cultivation/post-harvest treatment utilizing the effect of environmental conditions. Previous studies have reported differences in polyphenol content among strawberry cultivars [11,12] and some treatments [6,7]. However, there has been no comprehensive survey of specific pre- or post-harvest factors that enhance polyphenols. It is important to investigate the degree of abiotic stress factors that affect strawberry fruits during the processes of both cultivation and distribution. The objective of this study is to evaluate and optimize the environmental conditions that increase polyphenols in fruits towards understanding the mechanism of the polyphenol accumulation of strawberry. It also contributes to enhancing the value of strawberry products.

Targeting total polyphenol content in strawberry fruits, we first examined the typical values under different growing conditions at several developmental stages, then screened various treatment conditions during the pre-harvest and post-harvest periods. Focusing on a low-temperature treatment, the effect of which was confirmed by a statistical analysis, a transcriptome analysis was performed to identify the candidate pathways responsible for the increase in polyphenol content. Furthermore, the treatment was optimized for both preservation and functionality, and the degree of stress was determined based on changes in enzyme activity and abscisic acid content during the treatment period.

## 2. Results and Discussion

### 2.1. A Pre-Survey of the Basic Conditions during the Growth and Development of the Fruits

**Figure 1:**
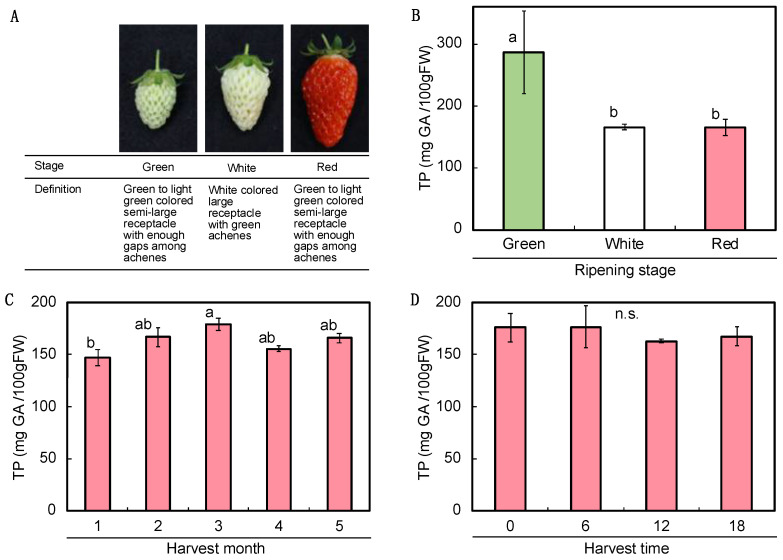
Total polyphenol contents in non-treated strawberry fruits during the ripening stages and under the different harvest conditions. Strawberry was grown by a container culture in a glass green house. Fruit ripening stages in strawberry (*Fragaria × ananassa*, cv. ‘Akihime’) were defined as Green, White, and Red (**A**). Total polyphenol contents in fruits were measured at different ripening stages (**B**), and various harvest months (**C**) or times (**D**). Different letters in each graph show significant (*p* < 0.05) differences with Tukey’s test (*n* = 4, r = 2).

The content ingredients of strawberries are influenced by growth and developmental stages. Therefore, basic data were first collected by fruit growth stage, harvest stage, and harvest time prior to pre- and post-harvest treatments. Fruit growth stages were defined as green, white, and red, as shown in Figure 1A. The average of the total polyphenol contents was distributed from 147 to 287 mg per 100 g of fresh weight of ripe fruit. These values were similar to those reported previously [12], suggesting that the selected method could be applied to the screening of polyphenol content in strawberries. The polyphenol content was the highest in the young green stage during fruit development (Figure 1B), which could be attributed to the richness of tannins, catechin, and ellagic acid in premature fruits [13]. These types of polyphenols have an antioxidant capacity in the plant body, and protect unripe seeds from herbivores and insect damage [14]. In terms of harvest month, the total polyphenol content appeared to increase as temperatures rose from midwinter and was predominantly richer in March than in January (Figure 1C). Previous trials from Japan on other varieties at different times also reported an increase from February onward compared to January [15]. Seasonal effects were found to positively regulate polyphenol content through temperature and light condition (light intensity and/or day length) [16,17]. As these month-to-month differences cannot be ignored, the pre-harvest treatments were concentrated in a short period of time, with separate controls for each treatment. There was no significant difference in total polyphenol contents among four harvesting times, at 0, 6, 12, and 18 o’clock (Figure 1D). This result did not limit the fruit harvest time used in the subsequent pre- and post- harvest trials.

### 2.2. Variation in Polyphenol Content under Different Pre- and Post-Harvest Conditions

First, we screened one to two samples from each experimental plot (Figure S1). Considering the variation within a treatment, we selected three for pre-harvest treatment, and four for post-harvest treatment (Table 1) [18,19]. Only one case showed significant differences in the total polyphenol content among the post-harvest experimental plots. 

Cold storage at 0 °C increased the total polyphenol content over time, with a significant difference between 10 and 0 days (non-stressed control). Strawberry fruits were not frozen at 0 °C [20]. Since ion leakage was not observed in strawberries frozen at −1 °C [21], physiological activity was maintained without reducing membrane fluidity due to cold injury. Polyphenol enrichment may be achieved by enzymes with sufficient activity and substrate affinity under cold conditions [22]. The United States Department of Agriculture reported that strawberry fruits can be stored for up to 7 d at 0 °C [20]. Cold stress stimulates the synthesis of polyphenols as antioxidants in many plant species such as lettuce, tomato, watermelon, and sweet potato [23,24,25].

### 2.3. Transcriptome Analysis to Verify the Conditions of Polyphenol Richness

**Figure 2:**
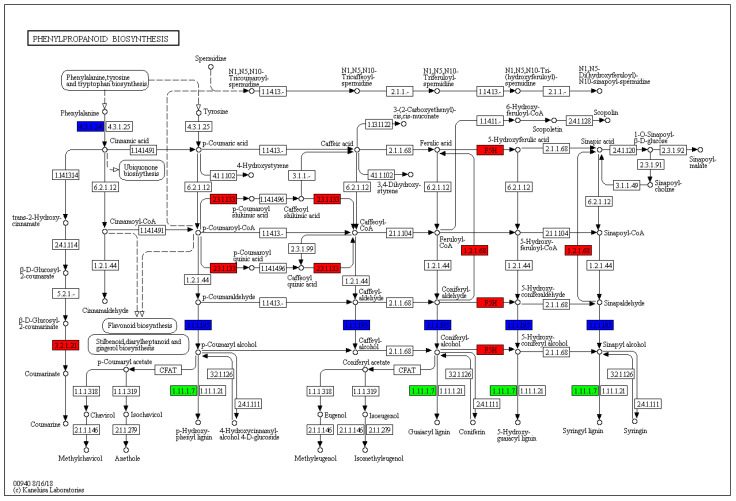
Phenylpropanoid biosynthesis pathway enriched by a post-harvest cold treatment in strawberry fruits. The enriched KEGG pathways are illustrated with copyright permission. Genes in red are upregulated, those in blue are downregulated, and those in green are either upregulated or downregulated.

A comprehensive gene expression analysis was conducted using RNAs extracted from post-harvest fruits before and after 10 days of cold treatment. After filtering the raw RNA-seq reads, the resulting clean reads were aligned with the reference genome of *F. vesca* [8]. Each sample generated approximately 50 million clean reads and 39 million clean reads (99% of the raw reads). Genome mapping rates ranged from 61 to 66%. Significant differentially expressed genes (DEGs) were determined according to the criteria of fold change > 2 and FDR (False Discovery Rate) < 0.05. A total of 201 genes were upregulated by cold treatment, while 253 genes were downregulated. A KEGG (Kyoto Encyclopedia of Genes and Genomes [26]) enrichment analysis clarified the significantly enriched pathways from both up- and downregulated DEGs (Table 2). Among these, we focused on phenylpropanoid biosynthesis (ko00940) because it is closely related to polyphenols as a precursor to biosynthesis and is widely distributed in plants [27]. Four upregulated DEGs encoding shikimate O-hydroxycinnamoyl transferase (EC [Enzyme Commission numbers]:2.3.1.133) and ferulate-5-hydroxylase (EC:1.14.-.-), coniferyl-aldehyde dehydrogenase (EC:1.2.1.68), and beta-glucosidase (EC:3.2.1.21); three downregulated DEGs encoding phenylalanine ammonia-lyase (EC:4.3.1.24), cinnamyl alcohol dehydrogenase (EC:1.1.1.195), and beta-glucosidase (EC:3.2.1.21); and three DEGs for both up (2 DEGs) and downregulated (1 DEG) encoding peroxidase (EC:1.11.1.7) were obtained (Figure 2). Phenylpropanoids, a diverse group of compounds derived from the carbon skeleton of phenylalanine, participate in plant defense, structural support, and survival [28]. Phenylpropanoid metabolites appear to contribute to the fruit ripening and fruit quality; they also have antioxidant capacities [29]. Similar relationships have also been observed in pear fruit regarding the relationship between cold storage and phenylpropanoid metabolism [30]. These results indicate that phenylpropanoid biosynthesis is one of the candidate pathways that contributes to the accumulation of total polyphenols in cold environments.

### 2.4. Improvement of Cold Storage Conditions

#### 2.4.1. Quality Preservation by Modifying Storage Methods

**Figure 3:**
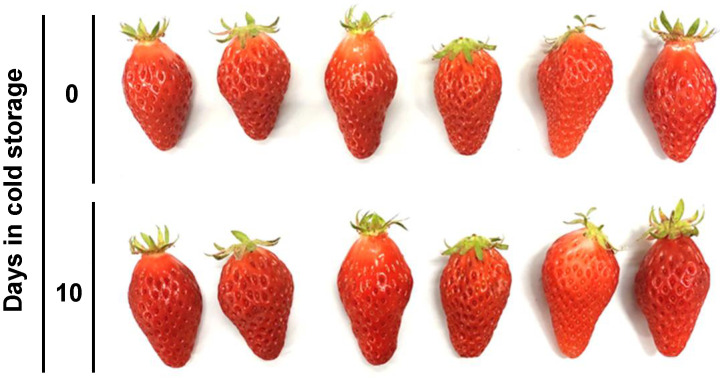
Difference in appearance of strawberry fruits before and after cold storage. Harvested strawberry fruits (upper) were kept at 0 °C for 10 days with porous film bag (lower). Photo represents six biological replications.

Ten days at 0 °C treatment was found to increase polyphenol content, which may include the effects of both cold and dry conditions. Low-temperature condition is a general storage method to prolong the post-harvest period of fruit, but strawberries are particularly susceptible to post-harvest storage because of their high respiration rate and susceptibility to moisture loss and pathogen development [31]. Weight loss and quality changes during fruit storage have also been observed in previous studies on strawberry [6,7]. This can prevent an accurate understanding of the physiological metabolism of strawberries [32]. Indeed, the weight of the fruits decreased as the days passed (by up to ~87% at 10 days, Table 3). To eliminate the influence of drought stress, we employed modified atmosphere packaging (MAP) using a porous film bag to preserve the quality of strawberry fruit by maintaining freshness [33]. Packaged strawberries showed little reduction in weight, Brix, and acidity, without significant differences during a 10-day storage period at 0 °C (Table 3). Furthermore, neither discoloration nor softening was observed (Figure 3). Consequently, MAP preserved the quality of strawberry fruit and was used for subsequent experiments.

#### 2.4.2. Polyphenol Contents and Antioxidant Activity in the Improved Cold Condition

**Figure 4:**
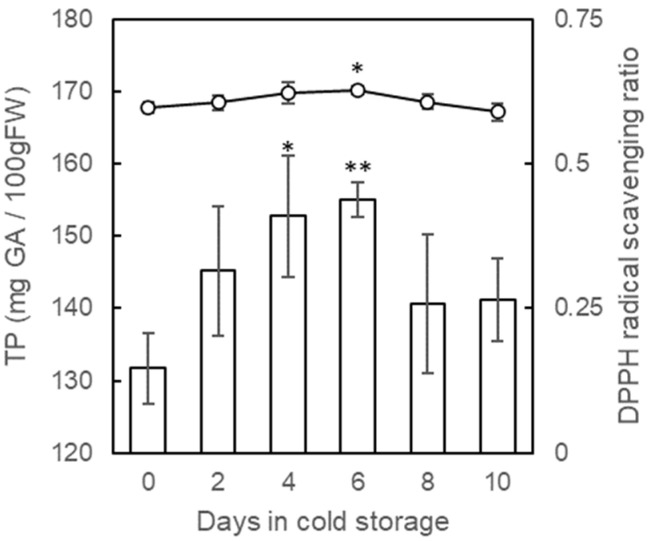
Relationship between total polyphenol contents and antioxidant activity (DPPH radical scavenging ratio) of strawberry fruits during cold storage. Total polyphenol contents and DPPH radical scavenging ratio are represented by bar and line graph, respectively. Harvested strawberry fruits were stored at 0 °C for 10 days with porous film bag. The vertical bar indicates a standard error of six replicates. Asterisks indicate significant differences in comparison to control (0 day) at *p* = 0.05 * or 0.01 ** with Wilcoxon test.

The polyphenol content of strawberries by MAP showed a significant increase on day 4 and 6 following cold storage (Figure 4). However, it decreased to the initial level after the day 8. This considerable gain of polyphenols might be due to the reduced damage to drought stress by MAP preservation. DPPH activity, which indicates antioxidant capacity, showed the same tendency as polyphenol content with significant improvement after 6 days of cold storage (Figure 4). Antioxidant activity in strawberry fruit is highly associated with polyphenol content [34]. Petriccione et al. [6] reported that the total phenolic content increased marginally until 3 days of cold storage at 2 °C and decreased toward the ninth day with changes in weight and quality. Similar results were obtained by Haung et al. [7], where both the total phenolic content and radical scavenging activity of DPPH in strawberry fruits increased to a peak on day 6 of storage at 0 °C, and then declined. In vegetative organs, such as the leaves and roots of strawberries, changes related to low-temperature acclimation increased throughout the cold acclimation period of 10 days [35,36]. This suggests that the acclimation mechanism to cold stimuli may occur transiently for 10 days. In the pre-harvest conditions, total polyphenol content did not show significant enhancement under a low-temperature atmosphere in the winter season (Table 1; harvest months January and February). There is still scope to examine artificial conditions, such as near 0 °C, for long days during the pre-harvest period. As stated above, PFB facilitated the optimization of the low-temperature treatment of polyphenol-rich strawberry fruits without a loss of quality and quantity.

#### 2.4.3. Stress Response to Low Temperature Based on Indicator Substances

**Figure 5:**
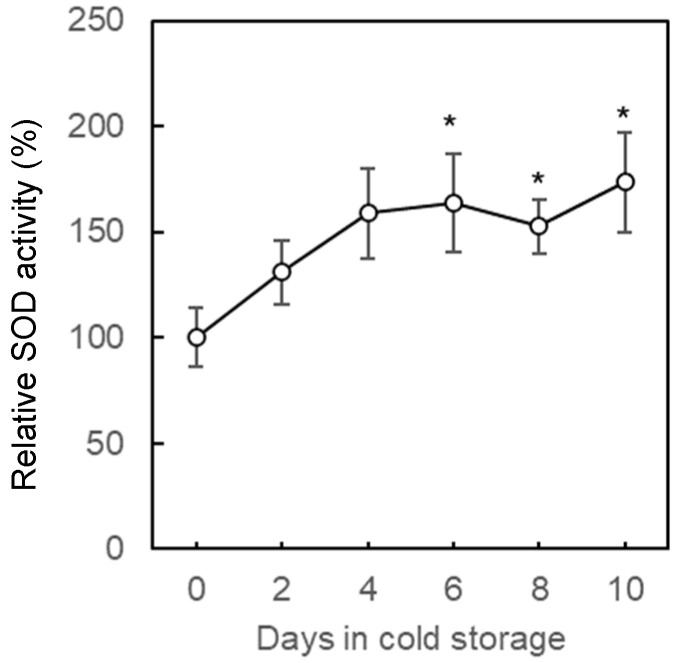
Changes in the superoxide dismutase (SOD) activity of strawberry fruits during cold storage. Harvested strawberry fruits were stored at 0 °C for 10 days with porous film bag. The vertical bar indicates a standard error of six replicates. Asterisks indicate significant differences in comparison to control (0 day) at *p* = 0.05 * with Wilcoxon test.

To evaluate the degree of stress under optimized cold storage conditions, known stress indicators were monitored continuously. Post-harvest oxidative stress occurs because of cold, water loss, or damage from disease during fruit storage, resulting in reactive oxygen species (ROS) such as H_2_O_2_, O_2_^−^ and OH^−^ radicals in the tissues [6,37]. The protection of fruit cells from oxidative injury by ROS depends on the levels of antioxidant enzymes, including superoxide dismutase (SOD) [38]. The activity level of SOD increased from the beginning to the end of cold storage and showed significant differences at day 6 and 10 compared to that at day 0 (Figure 5). Increased SOD activity has also been reported in studies on the antioxidant response of strawberry fruit [39]. Strawberry fruits respond to cold stress immediately, and the response reaches the climax around 6 days, which is consistent with the trend of polyphenol content.

**Figure 6:**
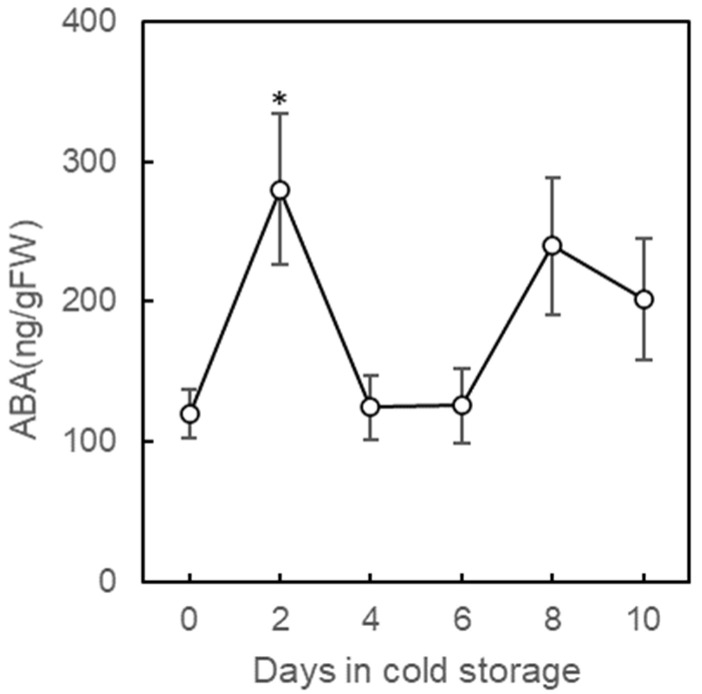
Changes in abscisic acid (ABA) contents of strawberry fruits during cold storage at 0 °C for 10 days. Harvested strawberry fruits were stored at 0 °C for 10 days with porous film bag. The vertical bar indicates a standard error of six replicates. Asterisks indicate significant differences in comparison to control (0 day) at *p* = 0.05 * with Wilcoxon test.

To ascertain the onset of the initial response, changes in ABA (abscisic acid) content were monitored during the cold storage period. ABA is a well-known mediator of stress-signaling pathways. In strawberries, ABA-dependent responses to both salt and drought stress increased the amount of phenylpropanoids, flavonoids, and ascorbic acid, together with upregulated gene expression [40]. A rapid accumulation of ABA was observed 2 days after cold storage (Figure 6). This accumulation of ABA may have induced the subsequent increase in polyphenols during day 4–6 and the gene expression of stress-responsive pathways, including phenylpropanoid synthesis, as identified by transcriptome analysis. From the second day onwards, the ABA decreased during day 4–6 and increased again on day 8–10. The second peak may be due to signs of drying stress caused by slight weight loss (Table 3).

## 3. Materials and Methods

### 3.1. Plant Material, Culture, and Treatment in a Pre-Survey of the Basic Conditions

‘Akihime’ was used as an experimental cultivar because both fruits and clone seedlings were easily obtained from the market. Plants for the pre-harvest treatment were grown in a glass greenhouse at Kobe University (Kobe, Japan), according to Ishibashi et al. [41]. Fruiting was promoted by the artificial pollination of flowers. The ripening stages were defined by the appearance, as shown in Figure 1. The fruits were harvested at different developmental stages (green, white, and red) by a color chart with a constant range. The approximate days from pollination to ripening were according to Ishibashi et al. [18]. To examine the successive changes in total polyphenol, fruits were harvested in different time periods (0, 6, 12, and 18 o’clock) and in different months (from January to May).

### 3.2. Screening of Effective Treatment in Pre- and Post-Harvest

Trials of pre-harvest treatment were conducted on fruits cultivated in a glass greenhouse at Kobe University. Those for post-harvest were obtained directly from agricultural producers in a glass greenhouse in Kobe, Japan, under similar environmental conditions in the same city as the university.

As pre-harvest treatments, ‘shading’ was applied by covering the fruits with aluminum foil at the white or green ripening stage and harvesting them when they turned red. ‘Wounding’ and ‘phytohormone’ treatments were conducted with red fruits. Wounding is made by making around a 3-mm cut of the fruit receptacle vertically or horizontally with a knife. Phytohormones were treated with 1-Naphthaleneacetic acid (NAA), gibberellin (GA), or abscisic acid (ABA) dissolved in the lanolin solution, according to Ishibashi et al. [18,41].

Post-harvest treatments for the screening of various stimuli were designed with different storage temperatures (0, 10, 20, 30, 40 and 50 °C) and days in storage (at 0 °C) for 0, 1, 5, and 10 days in containers without any cover. The ‘plasma’ treatment involved soaking in water with (+) or without (-) supplying plasma gases (O_2_, CO_2_, N_2_, air) administered for 7 min at 2 °C at a flow rate of 3 standard liters per minute. Indole-3-acetic acid (IAA), GA, or ABA was given by dissolving in water.

All the harvested or treated fruits were frozen in liquid nitrogen, ground into powder using Multi-beads Shocker (Yasui Kikai, Osaka, Japan), and stored at −80 °C until analysis.

### 3.3. Improve Cold Storage Methods (MAP) and Detailed Analysis

For detailed testing of the low-temperature storage conditions, the freshness-keeping package for vegetables and fruits (a porous film bag: P-plus, Sumitomo Bakelite, Tokyo, Japan) was adopted as modified atmosphere packaging (MAP). Fruits in the wrappings were stored at 0 °C for 0, 2, 4, 6, 8, and 10 days in the incubator.

### 3.4. Quantification of Total Polyphenol

The Folin–Ciocalteu method was used to determine total polyphenolic contents in frozen samples, according to the method described by Magalhães et al. [42] with slight modifications. Approximately 100 mg of frozen powder was homogenized in 10 times the volume of 90% (*v/v*) MeOH/0.5% acetate, incubated for 1 min, and then centrifuged at 20,400× *g* at 4 °C for 5 min. Subsequently, 50 µL of Folin–Ciocalteu phenol reagent (1:5, *v/v*; Merck KGaA, Darmstadt, Germany) and 6% (*w/v*) sodium carbonate solution were added to 50 µL of supernatant, mixed, and allowed to stand for 60 min at room temperature. A gallic acid (GA) standard curve was prepared from freshly prepared gallic acid (Wako Pure Chemical Industries, Osaka, Japan) solution in 90% (*v/v*) MeOH/0.5% acetate. Finally, absorbance was measured in a 96-well microplate at 750 nm using a grating microplate reader (SH-9000Lab; Corona Electric, Hitachinaka, Japan).

### 3.5. RNA Extraction

RNA was extracted automatically with a Maxwell 16 Automated Purification system (Promega, Madison, WI, USA), as described by Ishibashi et al. [43]. As a pre-treatment, 50–100 mg of frozen powdered sample was mixed with 500 µL of Fruit-mate (Takara Bio, Kusatsu, Japan). The mixture was vortexed and centrifuged at 13,000× *g* for 5 min. To 400 µL of supernatant, 200 µL lysis buffer was added to prepare the sample solution for extraction using the Maxwell purification system “RNA-PLANT” protocol. The resulting RNA samples were stored at –80 °C.

### 3.6. RNA-Seq Analysis

Biological repetitions with three totally independent RNAs were pooled for each treatment. The total bulk RNA was submitted to a custom service (GENEWIZ Japan, Kawaguchi, Japan) and was used for cDNA library preparation and sequencing. A standard cDNA library was prepared using polyA-selected mRNA. The cDNA libraries were sequenced on a HiSeq platform (Illumina, San Diego, CA, USA) in a 2 × 150 bp paired-end configuration. A bioinformatics analysis was performed using scientifically recognized algorithms by GENEWIZ Inc. The filtered data were subsequently aligned to the reference genome, *Fragaria vesca* v4.0.a1 [44]. The enrichment of the pathway was analyzed with the Kyoto Encyclopedia of Genes and Genomes (KEGG) database [45].

### 3.7. Determination of Antioxidant Capacity

DPPH (2,2-diphenyl-1-picrylhydrazyl) radical-scavenging activity was measured as described by Vicente et al. [46]. The frozen fruit powder was suspended in 1 mL of ethanol, incubated for 1 min, and then centrifuged at 20,400× *g* at 4 °C for 5 min. The supernatant was diluted 2–6 times and analyzed using DPPH Antioxidant Assay Kit (DOJINDO LABORATORIES, Kumamoto, Japan). Absorbance was measured at 492 nm using a grating microplate reader (SH-9000Lab; Corona Electric, Hitachinaka, Japan). The percentage of remaining DPPH against the extract volume was then plotted to obtain the amount of sample necessary to decrease the initial DPPH concentration by 50%, which was defined as the IC50. The DPPH radical scavenging ratio as the antioxidant activity of each sample was obtained from the percentage of remaining DPPH against the extract volume [7].

### 3.8. Measurement of Antioxidant Enzyme Activity

To measure the antioxidant activity of SOD, 1 g of frozen powder was suspended and stirred for 1 h in 4 mL of buffer (100 mM of potassium phosphate pH 7.8, 0.4 mM EDTA, 0.1% (*v/v*) Triton-X 100, 10 g/L PVPP). The homogenate was centrifuged at 20,400× *g* for 10 min, and the supernatant was used to assay the enzyme activity. All steps during the extract preparation were performed at 4 °C or on ice [46]. SOD activity was measured using the SOD Assay Kit-WST (Dojindo Laboratories, Kumamoto, Japan), following the method by Koyama et al. [47] with slight modifications. Absorbance was measured at 450 nm using a grating microplate reader (SH-9000Lab; Corona Electric, Hitachinaka, Japan). The 50% inhibition of SOD activity was calculated from the absorbance results.

### 3.9. Quantification of Abscisic Acid (ABA)

ABA was quantified using a plant hormone ABA ELISA Kit (CUSABIO, Houston, TX, USA). Approximately 100 mg of frozen powder was homogenized in sample extraction buffer. Crude extract was used for indirect ELISA with both rabbit-anti-ABA antibody and HRP-conjugated goat-anti-rabbit IgG antibody, according to the manufacturer’s protocol. Absorbance at 450 nm was measured using a grating microplate reader (SH-9000Lab; Corona Electric, Hitachinaka, Japan).

### 3.10. Statistical Analysis

The experiments were performed according to factorial design. Statistical analyses were performed using the JMP software (SAS Institute Japan Inc., Tokyo, Japan). The normality of each test was confirmed by the validity of the Shapiro–Wilk test. Subsequently, the data were analyzed by ANOVA, and the means were compared by each appropriate statistical method judged by normality and equal variances at a significance level of 0.01 and 0.05.

## 4. Conclusions

An exhaustive survey of changes in strawberry polyphenol content from pre-harvest and post-harvest conditions revealed that cold treatment at 0 °C was effective in boosting polyphenols. A pathway analysis with RNA-seq indicated the involvement of the phenylpropanoid synthesis pathway in enhancing polyphenol in a low-temperature environment. Modified atmosphere packaging clarified the simplified effect of cold stress on polyphenol, excluding drought stress. The stress indicators, such as antioxidant activity and abscisic acid, confirmed the proper timing of cold storage. These new findings will contribute to elucidating the post-harvest mechanisms for polyphenol accumulation and lead to the practical application of polyphenol-rich strawberries.

## Figures and Tables

**Table 1:** Total polyphenol contents in strawberry fruits under various pre- and post-harvest conditions.

Conditions			Total Polyphenol (mg GA/100 g FW)	
*Pre-harvest treatment*				
	Wounding	Control	176.0 ± 9.6	n.s.
		Vertical	205.1 ± 8.7	
	Phytohormone	Control	166.0 ± 13.1	n.s.
		NAA	155.7 ± 9.5	
*Post-harvest treatment*				
	Cold storage	Control (0 d)	150.7 ± 7.5	b
		0 °C for 1 d	170.2 ± 11.5	b
		0 °C for 5 d	178.7 ± 14.0	b
		0 °C for 10 d	198.3 ± 11.4	a
	Phytohormone	Control	136.7 ± 9.0	n.s.
		IAA	155.9 ± 11.7	
	Plasma	Control	141.7 ± 5.0	n.s.
		Air (−)	157.2 ± 5.8	
		Air (+)	148.2 ± 13.3	
		O_2_ (−)	151.7 ± 18.1	
		O_2_ (+)	147.6 ± 10.1	

Each value is expressed as mean ± standard deviation (*n* = 4, r = 2). Independent control plots were established for each treatment under the same condition. Different letters in the same row show significant (*p* < 0.05) differences with Tukey’s test. IAA: indole acetic acid; NAA: α-Naphthaleneacetic acid.

**Table 2:** Significantly enriched fifteen pathways from both up- and downregulated differentially expressed genes (DEGs) between control and cold treatment at 0 °C for 10 days in strawberry.

Pathway	Pathway	Category	DEGs *
ko00254	Aflatoxin biosynthesis	Metabolism	1
ko00261	Monobactam biosynthesis	Metabolism	1
ko00500	Starch and sucrose metabolism	Metabolism	7
ko04113	Meiosis—yeast	Cellular Processes	8
ko00940	Phenylpropanoid biosynthesis	Metabolism	10
ko03030	DNA replication	Genetic Information Processing	8
ko00982	Drug metabolism—cytochrome P450	Metabolism	6
ko00980	Metabolism of xenobiotics by cytochrome P450	Metabolism	7
ko00196	Photosynthesis—antenna proteins	Metabolism	2
ko00480	Glutathione metabolism	Metabolism	7
ko02025	Biofilm formation—*Pseudomonas aeruginosa*		1
ko04111	Cell cycle—yeast	Cellular Processes	8
ko04110	Cell cycle	Cellular Processes	8
ko00592	Alpha-linolenic acid metabolism	Metabolism	4
ko00550	Peptidoglycan biosynthesis	Metabolism	1

* DEGs with pathway annotation.

**Table 3:** General characteristics of strawberry fruits during cold storage with or without a modified atmosphere packaging (MAP).

Days in Cold Storage	Relative Fresh Weight (%)	Brix (%)	Acidity (%)
*Control (without MAP)*			
0	100.0 ± 0.0 a	-	-
5	94.2 ± 0.4 b	-	-
10	87.3 ± 1.0 c	-	-
*MAP*			
0	100.0 ± 0.0 n.s.	8.27± 0.36 n.s.	0.57 ± 0.03 n.s.
2	99.5 ± 0.2	8.43 ± 0.41	0.53 ± 0.02
4	99.4 ± 0.1	8.27 ± 0.48	0.52 ± 0.06
6	99.5 ± 0.1	8.83 ± 0.29	0.48 ± 0.02
8	99.3 ± 0.1	8.12 ± 0.18	0.49 ± 0.04
10	99.2 ± 0.2	7.90 ± 0.40	0.49 ± 0.02

Each value is expressed as mean ± standard error (*n* = 4–6). Different letters show significant (*p* < 0.05) differences with Tukey’s test.

## Data Availability

Not applicable.

## Supplementary Materials

The following supporting information can be downloaded at: https://www.mdpi.com/article/10.3390/plants11172220/s1, Figure S1: Relative total polyphenol contents in strawberry fruits under various pre- and post-harvest conditions..
