# Supplementary material for: Pre- and Post-Harvest Conditions Affect Polyphenol Content in Strawberry (Fragaria × ananassa)
Source: Plants (Basel). 2022 Aug 27;11(17):2220. doi: 10.3390/plants11172220 (PMC9460031; doi:10.3390/plants11172220)
Supplement: Supplementary file 1 [file plants-11-02220-s001.zip › plants-1795122-supplementary.pdf]

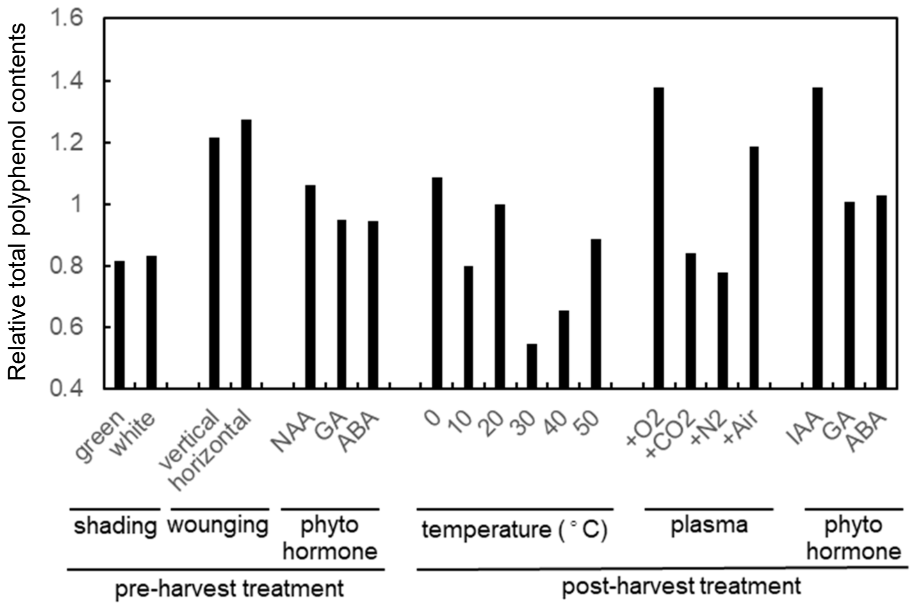

Figure S1. Relative total polyphenol contents in strawberry fruits under various pre- and post-harvest conditions. The bars are shown as relative value to the control in each treatment test (n=1 or 2).
